# Identification of Symptom Clusters and Core Symptoms in Inflammatory Bowel Disease: A Network Analysis in Chinese Cohorts

**DOI:** 10.1155/grp/4038190

**Published:** 2026-04-06

**Authors:** Zhihang Zhong, Yu Luo, Qin Xie, Xia Xie, Rong Zhao

**Affiliations:** ^1^ School of Nursing, Army Medical University/Third Military Medical University, Chongqing, China, tmmu.edu.cn; ^2^ Department of Gastroenterology, Southwest Hospital, Army Medical University, Chongqing, China, tmmu.edu.cn; ^3^ Department of Gastroenterology, Xinqiao Hospital, Army Medical University, Chongqing, China, tmmu.edu.cn; ^4^ Department of Gastroenterology, Army Medical Center of PLA/Daping Hospital, Army Medical University, Chongqing, China, tmmu.edu.cn

**Keywords:** exploratory factor analysis, inflammatory bowel disease, symptom cluster, symptom network

## Abstract

**Background:**

Research on symptom clustering in patients with inflammatory bowel disease (IBD) using network analysis is limited. Core symptom clusters in Chinese patients with IBD have not been clearly defined, and comparative evidence between Crohn′s disease (CD) and ulcerative colitis (UC) remains scarce, limiting the development of targeted symptom management strategies.

**Methods:**

Data were collected from three hospitals using the Chinese version of the symptom scale for IBD to assess symptom frequency, severity, and distress. Exploratory factor analysis was performed to identify symptom clusters. Symptom networks for CD and UC were constructed using JASP 0.19.1.0 to identify core and bridge symptoms. Bootstrap methods were applied to evaluate edge‐weight accuracy and the stability of centrality indices.

**Results:**

Abdominal pain (78.3%) was the most prevalent symptom in patients with CD, whereas mucopurulent bloody stool (75.2%) was most common in patients with UC. Five symptom clusters were identified. In CD, diarrhea (Rs = 1.700) emerged as the core symptom, whereas diarrhea (Rb = 1.812) and abdominal pain (Rb = 1.812) functioned as bridge symptoms. In UC, weight loss (Rs = 1.421) was the core symptom, with nutritional deficiency serving as the primary bridge symptom (Rb = 1.931). Bootstrap analysis showed narrow confidence intervals for edge weights, and the stability coefficients for strength and closeness centrality exceeded 0.25, indicating robust and reliable networks.

**Conclusion:**

Five distinct symptom clusters were identified, and separate symptom networks were established for CD and UC. These findings provide evidence for disease‐specific symptom prioritization and may support the development of targeted symptom management strategies.

## 1. Introduction

Inflammatory bowel disease (IBD), including ulcerative colitis (UC) and Crohn′s disease (CD), is a chronic inflammatory disorder of the digestive tract [1]. In recent years, IBD incidence has stabilized in North America and Western Europe [2], while rising sharply in emerging industrialized countries, including China [1]. This rise may be associated with lifestyle, dietary, and environmental changes driven by modernization [3].

IBD symptoms are complex and diverse, often appearing concurrently in “symptom clusters.” Up to 50% of IBD patients experience concurrent fatigue and pain [4], and abdominal pain and diarrhea often co‐occur with psychological symptoms, including anxiety and depression, affecting over 30% of IBD patients [5]. These symptoms lead to more doctor visits and complicate disease management [6].

Recent research on symptom clusters in IBD has revealed regional variations. European studies have identified symptom clusters involving fatigue, anxiety, and sleep disorders, whereas North American studies report clusters with abdominal pain, diarrhea, and stress. Latent class analysis has shown distinct symptom clusters, including high symptom burden and physical and psychological symptoms, influenced by factors such as sex, corticosteroid use, and disease activity. A prospective longitudinal study categorized IBD patients into three groups based on gastrointestinal and psychological symptoms, revealing that those with the highest symptom burden face greater risks of disease flare‐ups, treatment escalation, and increased medical resource use [7–10]. These findings suggest that regional variations in symptom clusters may result from differences in lifestyle, genetics, and cultural factors [11]. However, research on symptom clusters among Chinese IBD patients remains limited, creating a critical gap in understanding their disease experience.

This study is aimed at identifying and comparing the symptom clusters of UC and CD patients in China, revealing the different symptom patterns of the two types of IBD by analyzing the symptoms that often occur together, identify the core symptoms and bridge symptoms, and provide guidance for the development of efficient and targeted management.

## 2. Method

### 2.1. Study Design and Participants

This study was a multicenter, cross‐sectional study conducted at three tertiary hospitals in Chongqing, China. Patients were recruited from the IBD outpatient and gastroenterology inpatient departments. For inpatient recruitment, eligible patients were identified by the head nurse, who explained the study′s purpose, and after obtaining informed consent, two graduate students administered the survey. Inpatients were identified by the head nurse, who explained the study′s purpose. After obtaining informed consent, two graduate students administered the survey. Outpatients were identified by IBD specialists during their clinics, and after obtaining informed consent, the same graduate students administered the survey. All three hospitals treat IBD patients, with two having specialized IBD outpatient clinics. Identical inclusion and exclusion criteria were applied across three hospitals, following a standardized protocol to ensure consistency. The same survey instructions and administration procedures were used. Participants were informed that the survey was anonymous and their privacy would be fully protected.

### 2.2. Measures

This study used a general information questionnaire and the Chinese version of the symptom scale for inflammatory bowel disease (SCD‐IBD) [12]. The general information questionnaire collected participants′ age, sex, marital status, monthly income, education level, medical payment method, and disease type.

The SCD‐IBD scale consists of 18 items, each rated on a 5‐point Likert scale based on symptom frequency, severity, and distress. The item score is the average of the three ratings. The scale has an overall content validity index of 0.850, a Cronbach′s *α* coefficient of 0.900, and a test–retest reliability of 0.856, demonstrating high internal consistency and stability.

### 2.3. Samples

Exploratory factor analysis (EFA) was used to identify symptom clusters in patients with IBD. Following the commonly accepted guideline for factor analysis, which suggests a minimum of 10 samples per variable [13], the required sample size for this study is 18 variables × 10 samples = 180. To account for an estimated 10% survey attrition rate, at least 200 participants were recruited. This sample size is designed to satisfy the requirements of factor analysis and to ensure the stability and reliability of the results.

Recruitment took place between August and December 2024. The inclusion criteria were (a) diagnosed with inflammatory bowel disease, CD, or UC; (b) able to communicate clearly; and (c) willing to participate. The exclusion criteria were (a) having serious heart, lung, brain, or mental illnesses and (b) participating in other clinical studies.

### 2.4. Ethical Consideration

This study was approved by the hospital′s ethics committee, with Approval Number KY2024206.

### 2.5. Statistical Analysis

Data were analyzed using SPSS 27.0 and JASP (Version 0.19.1.0). Demographic characteristics and symptom data are presented as means, standard deviations (SDs), and proportions. Differences in prevalence rates were assessed using the chi‐square test.

EFA was performed on the total symptom composite score to identify the types and compositions of symptom clusters. Prior to EFA, symptom prevalence was examined using descriptive statistics.

Factors were selected based on the following criteria: (1) Eigenvalues of common factors greater than 1; (2) at least two symptoms with factor loadings ≥ 0.40; and (3) alignment of factor meanings with the relevant professional domain.

Network analysis was performed using JASP (Version 0.19.1.0). Each node represents a symptom, and edges indicate conditional independence relationships. Symptom centrality was evaluated using three metrics: strength (reflecting total direct connections), closeness (indicating centrality within the network), and betweenness (measuring the bridging role in shortest paths). Core symptoms were identified based on the highest centrality scores, whereas high‐prevalence symptoms with low centrality were considered sentinel symptoms.

Edge stability was assessed by calculating 95% confidence intervals (CIs) for edge weights via 1000 bootstrap samples. The stability of centrality metrics was evaluated through case‐dropping bootstrapping, with stability plots illustrating correlations as the proportion of retained cases decreased. A correlation above 0.5 was considered ideal, and 0.25 was the minimum acceptable threshold. Statistical significance for edge weights was set at two − tailed *α* = 0.05, with *p* < 0.05 considered significant.

## 3. Results

### 3.1. Reliability of the Sample Data

A total of 259 samples were collected, with a Cronbach′s *α* of 0.752, indicating good reliability.

### 3.2. Demographics and Characteristics of the Participants

A total of 259 IBD patients were included, comprising 134 patients with CD (51.7%) and 125 patients with UC (48.3%). The cohort included 140 males (54.1%) and 119 females (45.9%), with a mean age of 35.0 ± 15.9 years. The details are shown in Table 1.

**Table 1:** Demographics and characteristics of the participants (*n* = 259).

	Mean or *n*	%
Age	35.0 ± 15.9	
Gender		
Male	140	54.1
Female	119	45.9
Disease type		
CD	134	51.7
UC	125	48.3
Marital status		
Married	121	46.7
Unmarried	133	51.4
Others	5	1.9
Monthly household income per capita (RMB)		
< 3000	81	31.3
3000–5000	89	34.4
5000–10,000	64	24.7
> 10,000	25	9.7
Health care payment mode		
Military exemption	5	1.9
Employee medical insurance	64	24.7
Alternative medical coverage	140	54.1
Self‐payment	50	19.3
Education level		
Primary school and below	60	23.2
Junior high school	46	17.8
Senior high school	53	20.5
Associate degree college	69	26.6
Bachelor′s degree or higher	31	12.0

### 3.3. Symptom Prevalence, Frequency, Severity, and Distress

Symptom prevalence, frequency, severity, and distress are summarized in Table 2. Significant differences in symptom prevalence were observed between CD and UC patients, particularly for mucopurulent bloody stool and perianal symptoms (*p* < 0.001).

**Table 2:** Prevalence, frequency, severity, and distress of symptoms.

Symptom	Prevalence (%)				Frequency (*m* *e* *a* *n* ± *S* *D*)	Severity (*m* *e* *a* *n* ± *S* *D*)	Distress (*m* *e* *a* *n* ± *S* *D*)
	Total (*n* = 259)	CD (*n* = 134)	UC (*n* = 125)	*p*			
DI (diarrhea)	64.4	67.9	60.8	0.147	3.17 ± 1.376	3.001 ± 1.414	2.846 ± 1.47
AP (abdominal pain)	72.6	78.3	66.4	0.068	3.309 ± 1.302	3.031 ± 1.272	2.988 ± 1.342
AD (abdominal distension)	51.8	57.4	45.6	0.603	2.691 ± 1.405	2.452 ± 1.291	2.421 ± 1.319
MB (mucopurulent bloody stool)	61.0	47.7	75.2	< 0.001	2.68 ± 1.324	2.463 ± 1.295	2.367 ± 1.3
TE (tenesmus)	58.5	51.9	63.2	0.362	2.915 ± 1.384	2.761 ± 1.416	2.486 ± 1.31
PA (perianal abscess)	30.9	50.8	9.6	< 0.001	1.888 ± 1.144	1.873 ± 1.189	1.803 ± 1.163
AF (anal fissure)	12.3	21.7	2.4	< 0.001	1.502 ± 1.065	1.409 ± 0.97	1.378 ± 0.934
FI (fistula)	19.0	34.4	1.6	< 0.001	1.541 ± 1.035	1.471 ± 0.933	1.51 ± 1.021
ND (nutritional deficiency)	49.8	49.2	50.4	0.995	2.506 ± 1.39	2.405 ± 1.409	2.228 ± 1.346
WL (weight loss)	54.9	54.5	55.2	0.611	2.745 ± 1.435	2.568 ± 1.454	2.375 ± 1.456
AN (anemia)	44.1	47.7	40.0	0.013	2.259 ± 1.269	2.097 ± 1.176	1.934 ± 1.155
SL (skin lesions)	20.8	23.2	18.4	0.233	1.537 ± 0.935	1.502 ± 0.933	1.494 ± 0.982
OU (oral ulcers)	23.9	27.6	20.0	0.031	1.817 ± 1.136	1.757 ± 1.154	1.703 ± 1.096
OM (ocular manifestations)	4.9	8.3	1.6	0.535	1.132 ± 0.604	1.155 ± 0.614	1.132 ± 0.536
FA (fatigue) [14]	56.2	55.2	57.6	0.987	2.367 ± 1.158	2.205 ± 1.148	2.104 ± 1.138
AX (anxiety)	44.1	52.3	35.2	0.074	2.293 ± 1.147	2.116 ± 1.176	2.046 ± 1.037
DE (depression)	17.0	18.6	15.2	0.546	1.479 ± 0.804	1.456 ± 0.831	1.386 ± 0.703
SD (sleep disturbance)	31.0	27.6	34.4	0.052	2.293 ± 1.248	2.1 ± 1.206	2.069 ± 1.265

### 3.4. Symptom Clusters Based on Symptom Scores

Seventeen symptoms with a total prevalence greater than 10% were included in the EFA. Ocular manifestations (OM), with a prevalence of 4.9%, were excluded prior to EFA, in line with previous studies that applied prevalence‐based criteria for symptom selection, as low‐prevalence symptoms may compromise the stability of the factor structure [15, 16].

**Figure 1:**
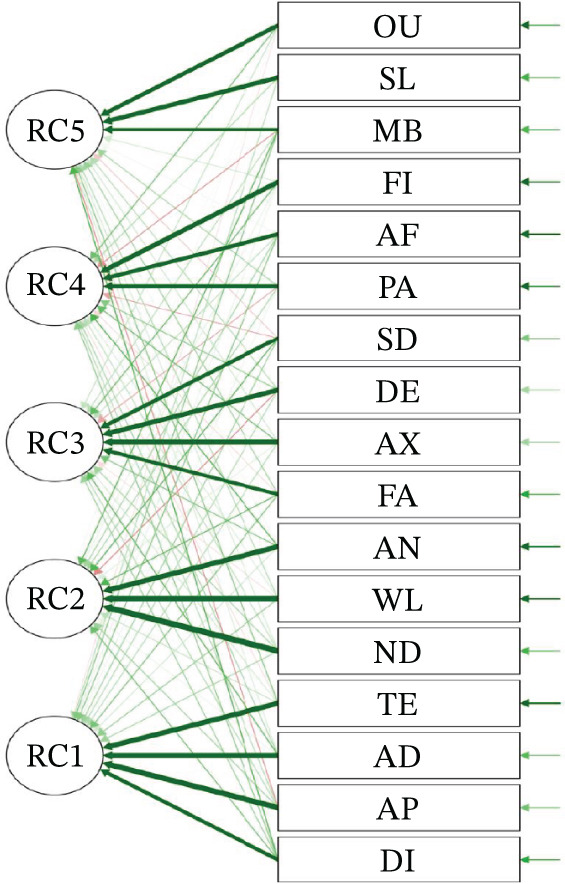
Pathway diagram of IBD symptoms.

**Figure 2:**
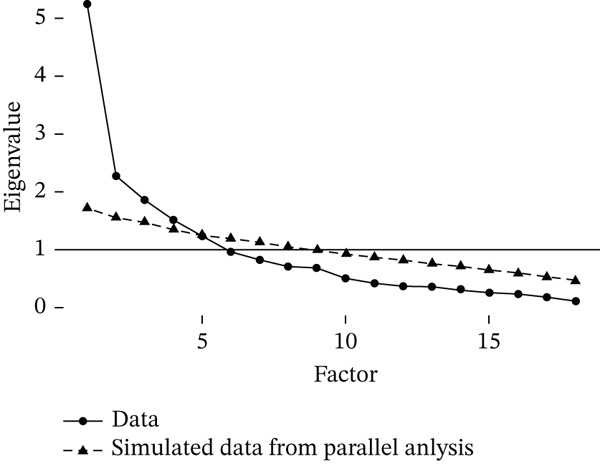
Screen plot of IBD symptoms.

The Kaiser–Meyer–Olkin (KMO) value was 0.733, and Bartlett′s test of sphericity was significant (*p* < 0.001), confirming the suitability of the data for factor analysis. Five factors with eigenvalues greater than 1 were extracted via principal axis factoring and varimax rotation, which explained 67.0% of the cumulative variance. The scree plot and path diagram supported the five‐factor model (Figures 1 and 2). Factor loadings are shown in Table 3.

**Table 3:** Factor loadings of IBD patients (*n* = 259).

Symptoms	Gastrointestinal symptoms	Nutrition‐related symptoms	Psychological symptoms	Perianal disease	Systemic inflammatory manifestations	Uniqueness
AP	0.833					0.223
TE	0.802					0.299
AD	0.800					0.263
DI	0.666					0.357
WL		0.895				0.144
ND		0.886				0.153
AN		0.806				0.212
AX			0.795			0.281
DE			0.768			0.307
SD			0.677			0.410
FA			0.662			0.411
FI				0.757		0.355
PA				0.713		0.426
AF				0.698		0.442
SL					0.748	0.410
OU					0.711	0.434
MB					0.596	0.487
Variance contribution rate (%)	0.158	0.155	0.143	0.115	0.099	
Cumulative variance contribution rate (%)	0.158	0.313	0.456	0.571	0.670	

*Note:* The rotation method applied was varimax.

Factor labels were assigned by two graduate students (Z.H., Q.X.), with final validation by the supervising professor (Y.L.). The labeling process was based on symptom co‐occurrence patterns, clinical relevance, and the common clinical presentation of symptoms within each factor. These labels reflect symptom clusters summarizing key symptoms observed in IBD patients.

The gastrointestinal cluster included abdominal pain, diarrhea, abdominal distension, and tenesmus, reflecting common symptoms of intestinal dysfunction and inflammation.

The nutrition‐related cluster comprised weight loss, nutritional deficiency, and anemia, highlighting the metabolic consequences of chronic inflammation and malabsorption, commonly seen in both UC and CD patients.

The psychological cluster included anxiety, depression, sleep disturbance, and fatigue, reflecting the psychological burden often experienced by IBD patients.

The perianal disease cluster, which included anal abscesses, anal fistulas, and anal fissures, was associated with the transmural inflammation characteristic.

The systemic inflammatory manifestation cluster included skin lesions, oral ulcers, and mucopurulent bloody stool, reflecting extraintestinal symptoms that are often linked to systemic disease activity in IBD.

### 3.5. Network Analysis

**Figure 3:**
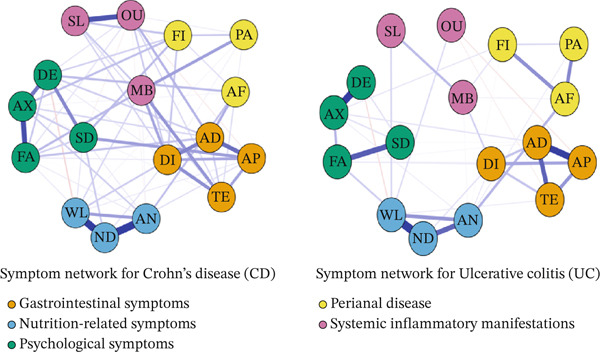
Symptom network for Crohn′s disease and ulcerative colitis.

In the symptom networks (Figure 3), gastrointestinal symptoms formed densely connected clusters, indicating strong co‐occurrence relationships. Abdominal pain showed close associations with diarrhea and abdominal distension, suggesting a central role within the gastrointestinal symptom cluster.

In CD, perianal conditions formed a relatively independent but internally cohesive module, with limited connections to other symptom domains. In contrast, in UC, psychological symptoms constituted a distinct module that was moderately connected to fatigue, indicating partial integration with systemic symptom burden.

Nutritional symptoms formed tightly connected clusters in both CD and UC patients and showed moderate connectivity with the broader symptom network, suggesting that nutritional impairment represents a shared but not central symptom domain across the two diseases.

In the UC network, mucopurulent bloody stool emerged as a key gastrointestinal‐related symptom that was strongly connected to diarrhea and other gastrointestinal symptoms. Although it showed indirect associations with nutritional and psychological symptoms, these connections were comparatively weaker, indicating a more localized role within the gastrointestinal domain rather than a global bridging function.

### 3.6. Centrality Analysis Results

**Figure 4:**
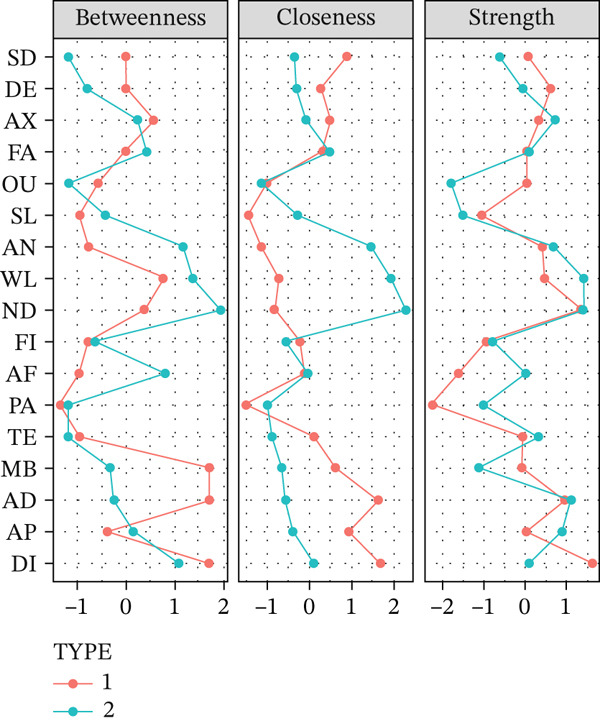
Centrality analysis of Crohn′s disease and ulcerative colitis (1 = Crohn^′^s disease, 2 = ulcerative colitis).

In this centrality analysis, strength centrality was interpreted as the extent of direct symptom associations, closeness reflected how centrally a symptom was positioned within the overall network, and betweenness indicated a potential role in connecting different symptom domains. The results of centrality analysis (Figure 4) revealed that in patients with CD, diarrhea, abdominal distension, and nutritional deficiency had the highest strength centrality scores, indicating that these symptoms were directly connected to multiple other symptoms and may have a broader influence on symptom co‐occurrence. In UC, nutritional deficiency, weight loss, and anemia also showed the highest strength centrality, indicating that nutrition‐related symptoms were widely and directly connected to other symptoms across the network.

### 3.7. Network Stability

**Figure 5:**
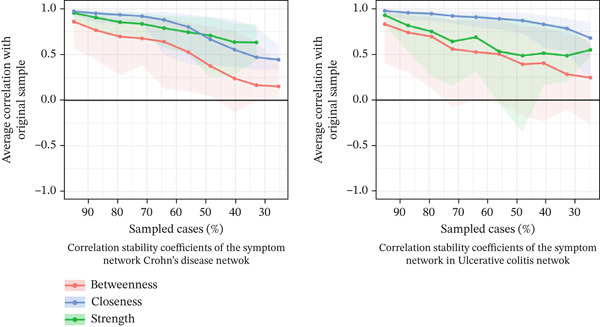
Bootstrap analysis results of edge weights in Crohn′s disease and ulcerative colitis.

Bootstrap analysis (Figure 5) showed that most major edges within gastrointestinal symptom clusters in both CD and UC networks had relatively narrow CIs, indicating stable estimation of key associations. Strong gastrointestinal symptom links, including those between diarrhea and abdominal distension, were consistently observed across bootstrap samples, whereas some peripheral connections showed greater uncertainty.

**Figure 6:**
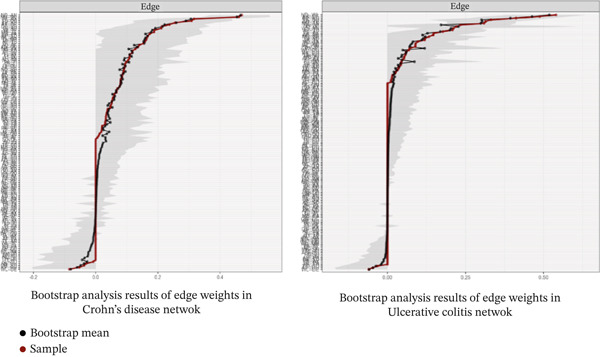
Correlation stability coefficients of the symptom network in Crohn′s disease and ulcerative colitis.

Correlation stability (CS) analysis (Figure 6) indicated that strength centrality demonstrated good stability in both diseases, remaining above recommended thresholds when up to 40% of cases were removed. Closeness centrality showed lower but acceptable stability, remaining above minimum thresholds at a 30% case retention level.

These results support the interpretation of findings based on strength centrality and major symptom associations.

## 4. Discussion

This study employed EFA to identify symptom clusters in Chinese patients with IBD and used network analysis to compare the symptom network structures between CD and UC. The results show that IBD symptoms do not co‐occur randomly, but rather form disease‐specific modular structures with key bridging symptoms, revealing coupling patterns between symptoms in CD and UC.

### 4.1. Symptom Clusters in IBD Patients

Five symptom clusters were identified in patients with IBD. Gastrointestinal symptoms represent the primary reason for healthcare utilization and remain central therapeutic targets, consistent with prior research [10]. Nutrition‐related symptoms reflect the common nutritional burdens and functional impacts experienced by IBD patients. The psychological cluster encompasses the common psychological and sleep burdens in IBD patients, with previous research suggesting that psychological distress may be related to symptom experience [17]. The perianal disease cluster was more commonly observed in CD patients and represents a well‐recognized clinical feature of CD, aligning with prior reports on perianal complications [18, 19]. The systemic inflammatory manifestation cluster involves extraintestinal manifestations related to inflammatory activity.

### 4.2. Core Symptom Network in UC and CD

Network analysis revealed clear structural differences between CD and UC. The CD symptom network was more densely interconnected, whereas the UC network exhibited a more modular structure with clearer boundaries between clusters, suggesting differences in cross‐system symptom coupling.

In CD, the overall network centers around gastrointestinal symptoms. Diarrhea has the highest centrality, with high closeness and strength, indicating that it is not only prevalent but also occupies a central position within the symptom network. Abdominal pain also shows high centrality (Rs = 1.043), emphasizing its prominence in the CD network. These findings are consistent with previous studies identifying diarrhea as a core symptom associated with intestinal dysfunction and impaired absorption in CD [20, 21]. Perianal symptoms formed a distinct cluster in both EFA and network visualization, reflecting the clinical features of CD [18, 19]. However, their relatively low centrality, particularly for perianal abscess, suggests that these symptoms may constitute a more self‐contained subnetwork, exerting a limited influence on the broader symptom structure.

In UC, the network was primarily organized around nutrition‐related symptoms. Nutritional deficiency and weight loss exhibit high centrality in multiple indices (Rs = 1.413 and 1.421, respectively), with the edge between WL‐ND being particularly strong, indicating a robust coupling between these two symptoms. Gastrointestinal symptoms form a distinct localized cluster, with AD‐AP and AD‐TE showing strong coupling. However, the dominance of gastrointestinal symptoms in the overall network is lower than that of nutrition‐related symptoms, suggesting that nutritional impairment plays a more central role in the UC symptom network. This finding aligns with previous studies highlighting the clinical significance of nutrition in UC [22].

An important observation in the UC symptom network is the discrepancy between symptom prevalence and network centrality. Although mucopurulent bloody stool is highly prevalent among UC patients (75.2%), its strength and expected influence were low and negative (Rs = −1.136) in the standardized network. This indicates that, within the estimated cross‐sectional network, this symptom occupies a relatively peripheral structural position despite its clinical prominence. Negative centrality values reflect relative network positioning and do not imply inverse or protective clinical effects. Although some previous studies have described highly prevalent but low‐centrality symptoms as “sentinel symptoms” [18, 23], the present cross‐sectional analysis does not support such a role for mucopurulent bloody stool. Longitudinal studies are needed for further validation.

### 4.3. Bridge Symptoms in CD and UC

Bridge symptoms, characterized by high betweenness centrality, represent symptoms positioned between clusters and reflect pathways through which different symptom domains are interconnected [19].

In CD patients, diarrhea, abdominal pain, and mucopurulent bloody stool had high betweenness centrality (Rb = 1.812, 1.812, and 1.625, respectively), suggesting that these symptoms occupy important positions linking distinct symptom domains. This pattern aligns with the established clinical significance of gastrointestinal symptoms in CD [24, 25]. In UC, nutritional deficiency, weight loss, and anemia showed high betweenness centrality, indicating that nutrition‐related symptoms function as bridges connecting multiple symptom domains. Previous studies have shown that persistent inflammation and metabolic burden contribute to adverse nutritional outcomes in IBD patients [26, 27], and anemia is commonly linked to fatigue and other functional symptoms [28, 29]. Although diarrhea also functioned as a bridge symptom in UC, nutrition‐related symptoms appeared to have a more prominent bridging effect, reflecting the different patterns of symptom interconnection between CD and UC [30, 31].

### 4.4. Implications for Symptom Management and Prevention Strategies

This study provides a theoretical framework for symptom management strategies informed by symptom co‐occurrence networks. Symptoms with high centrality or bridging positions may warrant particular attention in symptom assessment and monitoring, as changes in these symptoms could be associated with broader alterations in the symptom network.

In CD, gastrointestinal symptoms, particularly diarrhea, demonstrated prominent positioning within the symptom network and may merit close clinical observation. In UC, nutrition‐related symptoms and anemia occupied central and bridging positions, underscoring the importance of routine nutritional assessment and anemia screening as part of comprehensive symptom evaluation. [32–34].

Previous studies have explored interventions targeting inflammation control, nutritional support, and psychological or sleep‐related symptoms in IBD [30, 31, 35–39]. These studies provide a clinical context for interpreting the symptom interconnections observed in the present analysis. Further longitudinal and interventional studies are needed to determine whether targeting central or bridge symptoms can meaningfully alter symptom trajectories or improve clinical outcomes. Until such evidence is available, the results should be viewed as exploratory and informative for future research. In addition, it should be noted that the symptom network structures identified in this study may also be influenced by underlying biological processes. Although biomarker data were not available in the present study, previous studies have suggested that inflammatory biomarkers, including plasma cytokines, may be related to overall symptom burden in IBD [40–43]. Future studies integrating clinical symptom networks with inflammatory biomarkers may help further elucidate these relationships.

### 4.5. Limitations

This study has several limitations. First, the sample was drawn from three hospitals in Chongqing, China, which may limit the generalizability of the findings to other regions or populations. Second, the cross‐sectional design does not capture temporal changes in symptom networks related to disease progression or treatment effects. Longitudinal studies are therefore needed to examine the dynamic evolution of symptom networks. Finally, the lack of biomarker data prevented assessment of the relationship between symptom network structures and underlying biological processes [44–47]. Future studies integrating clinical symptom networks with inflammatory biomarkers, such as plasma cytokines, may provide deeper insight into the biological basis of symptom co‐occurrence.

## 5. Conclusion

This study used EFA and network analysis to identify five symptom clusters in Chinese IBD patients and construct distinct symptom networks for CD and UC patients, highlighting differences in symptom propagation. The findings provide insights for symptom management and precision treatment while facilitating comparisons with international research.

**Nomenclature**
IBD: inflammatory bowel disease
UC: ulcerative colitis
CD: Crohn′s disease
SCD‐IBD: symptom scale for inflammatory bowel disease
EFA: exploratory factor analysis
KMO: Kaiser–Meyer–Olkin
DI: diarrhea
AP: abdominal pain
AD: abdominal distension
MB: mucopurulent bloody stool
TE: tenesmus
PA: perianal abscess
AF: anal fissure
FI: fistula
ND: nutritional deficiency
WL: weight loss
AN: anemia
SL: skin lesions
OU: oral ulcers
OM: ocular manifestations
FA: fatigue
AX: anxiety
DE: depression
SD: sleep disturbance

## Author Contributions

Z.Z. analyzed and interpreted the data and made the primary contributions to manuscript drafting. Y.L. made significant contributions to the conception, design, and revision of the manuscript. Q.X., X.X., and R.Z. contributed to the questionnaire survey and data collection.

## Funding

No funding was received for this manuscript.

## Disclosure

All authors have read and approved the final manuscript.

## Ethics Statement

The study was approved and authorized by the ethics committees of various participating hospitals (Approval # KY2024206, First Affiliated Hospital of Army Medical University, the leading site). Informed consent was obtained from all individuals included in this study.

## Consent

The authors have nothing to report.

## Conflicts of Interest

The authors declare no conflicts of interest.

## Data Availability

The datasets generated and/or analyzed during the current study are not publicly available due to data confidentiality, but are available from the corresponding author on reasonable request.
